# Reduced fitness of Atlantic salmon released in the wild after one generation of captive breeding

**DOI:** 10.1111/eva.12028

**Published:** 2012-11-22

**Authors:** Emmanuel Milot, Charles Perrier, Lucie Papillon, Julian J Dodson, Louis Bernatchez

**Affiliations:** 1 Département de Biologie, Institut de Biologie Intégrative et des Systèmes (IBIS), Université LavalQuébec City, QC, Canada; 2 Départment de biologie, Pavillon Vachon, Université LavalQuébec City, QC, Canada

**Keywords:** contemporary evolution, evolutionary change, fitness, parentage analysis, salmonid, supportive breeding

## Abstract

Salmonids rank among the most socioeconomically valuable fishes and the most targeted species by stocking with hatchery-reared individuals. Here, we used molecular parentage analysis to assess the reproductive success of wild- and hatchery-born Atlantic salmon over three consecutive years in a small river in Québec. Yearly restocking in this river follows a single generation of captive breeding. Among the adults returning to the river to spawn, between 11% and 41% each year were born in hatchery. Their relative reproductive success (RRS) was nearly half that of wild-born fish (0.55). RRS varied with life stage, being 0.71 for fish released at the fry stage and 0.42 for fish released as smolt. The lower reproductive success of salmon released as smolt was partly mediated by the modification of the proportion of single-sea-winter/multi-sea-winter fish. Overall, our results suggest that modifications in survival and growth rates alter the life-history strategies of these fish at the cost of their reproductive success. Our results underline the potential fitness decrease, warn on long-term evolutionary consequences for the population of repeated stocking and support the adoption of more natural rearing conditions for captive juveniles and their release at a younger stage, such as unfed fry.

## Introduction

Supplementation of wild populations with captive-bred individuals is a common practice, particularly so in salmonid species. However, such practices have been widely criticized for both their low efficiency in preventing wild population declines and their potential contribution to fitness reduction (Aprahamian et al. 2003; Araki et al. 2008; Fraser 2008; McClure et al. 2008). Even though captive breeding practices have been improved, for example, through the release of young individuals (i.e., fry or smolt) produced using yearly caught local wild breeders, to minimize domestication and loss of local adaptation, significant fitness decrease in hatchery-reared relative to wild fish persists (Araki et al. 2007b, 2009; Thériault et al. 2011; Anderson et al. 2012; Christie et al. 2012). The achievement of successful management and conservation practices thus requires documenting the fate of hatchery-reared individuals released in the wild.

Different selection regimes in hatcheries than in the wild may result in relatively low reproductive success of hatchery-reared fish released in the wild (Fleming and Einum 1997; McGinnity et al. 2003; Araki et al. 2007b, 2009; Hutchings and Fraser 2008; McLean et al. 2008; Thériault et al. 2010, 2011). First, nonrandom sampling or artificial breeding can cause unintentional selection on particular traits. Second, the disruption of natural mate choice and hence of sexual selection mechanisms may have important genetic implications for the next generation (Wedekind 2002; Neff et al. 2008; Anderson et al. 2012). Third, mortality patterns and hence viability selection during life in the hatchery may differ from the wild (Ford 2002; Mignon-Grasteau et al. 2005; Frankham 2008). Therefore, selection may decrease the fitness of hatchery-reared fish once they are released in the wild (Araki et al. 2007b; Thériault et al. 2011). Moreover, Christie et al. (2012) showed that the lifetime reproductive success of first-generation hatchery steelhead was twice higher than that of wild-born fish bred in hatchery, which was a clear demonstration of adaptation to captivity. Ultimately, heritable traits selected in the hatchery that are disadvantageous in nature may have long-lasting evolutionary impacts on the fitness of wild-stocked populations via introgressive hybridization (McGinnity et al. 2003; Araki et al. 2008).

Captive rearing can also induce plastic phenotypic responses that may result in environmental carryover effects on their life-history traits once released in the wild. Carryover effects have been defined as the nonlethal influence on individual performance of a previous (environmental) event in its life (Harrison et al. 2011). Hatchery and wild environments differ by many aspects including fish density, spatial and temporal heterogeneity of physical environment, intra- and interspecific interactions, and food availability that may lead to plastic phenotypic divergence among wild and captive fish, including growth rate, morphology, and behavior (Metcalfe et al. 2003; Kostow 2004; Blanchet et al. 2008; Hawkins et al. 2008). For instance, hatchery-reared salmonids fed *ad libitum* generally have higher growth rates than wild conspecifics (McDonald et al. 1998; Berejikian et al. 2000). Such modifications may later affect the life history of stocked salmonids, notably a reduction in their average number of winters at sea compared with wild-born fish (Kostow 2004; Jonsson and Jonsson 2006; Kallio-Nyberg et al. 2011; but see McGinnity et al. 2003). Ultimately, their reproductive success in the wild may be affected (Weir et al. 2005; Thériault et al. 2010, 2011) partly because spawner body size is affected by time spent at sea and correlates with fecundity (Fleming 1996). Thériault et al. (2011) notably showed that the reproductive success of coho salmon (*Oncorhynchus kisutch*) released as fry was similar to that of wild fish, whereas salmon released as smolts performed worse, suggesting important carryover effects of hatchery environment on the reproductive success of smolt-stocked fish. The wild progeny of such stocked individuals may moreover also experience a reduced reproductive success originating from genetic carryover effects (Araki et al. 2009). Introgressive hybridization could thus magnify these effects and result in negative evolutionary change on wild populations.

Atlantic salmon (*Salmo salar*) is one of the most socioeconomically important fish species, through recreational fishing, fisheries, and aquaculture. Over the last decades, wild Atlantic salmon populations have been widely impacted by various anthropogenic pressures, resulting in the common use of supportive breeding to compensate decreasing recruitment (Aprahamian et al. 2003; Jonsson and Jonsson 2009). Here, we studied a small anadromous Atlantic salmon population spawning in the Malbaie River, Québec, which has been subjected for 20 years to a supportive breeding program releasing fry and smolt fish produced using local adults yearly caught in the river. We used molecular parentage analyses to quantify and compare the reproductive success in the wild of wild-born and of fry and smolt-stocked hatchery-born Atlantic salmon. We thus tested three hypotheses: (i) hatchery-reared individuals will have a lower reproductive success relative to wild-born fish because of detrimental selective and carryover effects of the hatchery environment, (ii) fish stocked as fry will have a higher reproductive success than those stocked 1 year later as smolt, as a result of stronger selective and environmental carryover effects in the latter, and (iii) smolt-stocked salmon returning to spawn will differ from wild and fry-stocked fish in their proportion of multi-sea-winter fish (MSW) as an effect of longer time spent in hatchery on their subsequent life history. We then discuss the potential role of mechanisms such as selection and environmental carryover effects of a single generation of captive breeding in causing the observed fitness reduction in hatchery-born salmon released in the wild and the implications for stocking practices.

## Materials and methods

### Study site

The Malbaie River is located on the North shore of the St. Lawrence estuary, Québec, Canada (47°67′N; 70°16′W), and harbors a small anadromous spawning population of Atlantic salmon. On average, 283 adult spawners annually returned to the river from 1997 to 2004 (Table S1), a number reaching only 20% of the appropriate conservation threshold for this population (Minister of Natural Resources and Wildlife, Québec). A dam acting as a complete barrier to upstream migration is located eight kilometers upstream to the river mouth. During the upstream migration from the St. Lawrence to the Malbaie River, adult fish are trapped in a cage and transported just above the dam to be released upstream. Spawning grounds and suitable habitats for Atlantic salmon juveniles extend over a stretch starting from the dam and ending 35 km upstream.

### Supportive breeding

Supportive breeding began in 1992 when a number of returning adults were first caught at the dam and transferred to the provincial hatchery at Tadoussac, located approximately 60 km downstream of the Malbaie River estuary along the St. Lawrence River. The pool of breeders coming from the wild has been maintained at almost 20 individuals, about half of them being renewed each year by adding new breeders caught at the dam. No fish born in the hatchery was kept all its life in captivity and used as a captive breeder. However, there is a possibility that some returning adult trapped at the dam and brought to the hatchery each year to maintain the pool of breeders were themselves hatchery-born fish that had been released as fry or smolt in the Malbaie River during supportive breeding efforts. Breeders were mated in captivity and the progeny reared in indoor tanks at density of about 500 fish/m^3^ until being stocked either as fry or smolt, respectively, after four and fifteen months following hatching. From 1992 to 2002, a total of 133 579 eggs, 890 489 fry and 323 923 smolts have been stocked on the spawning grounds of the Malbaie River (Table S1).

### Sampling

Scale and fin samples were taken on all anadromous adults returning to the river between 2002 and 2004, as well as on all breeders at the Tadoussac hatchery. The sex of 69% of the fish was determined with some confidence using secondary sexual characters when possible (Maisse and Bagliniere 1986). Age, that is, number of years in river + number of years at sea, was determined from scale growth patterns (Lund and Hansel 1991; Stokesbury and Lacroix 1997). Scale growth patterns also allowed identifying returning adults that were born in hatchery and released at the smolt stage (Auclair E., Bernatchez L., Dodson J.J., unpublished data). During the summers of 2003, 2004, and 2005 and before the stocking of hatchery-born fry each year, fry born in the river were randomly sampled using electrofishing over a stretch starting from the dam and ending 35 km upstream. All 136 breeders present at the Tadoussac hatchery from 2000 to 2002, all of the 876 returning adults that were caught in 2002, 2003, and 2004, and 1141 fry caught in 2003, 2004, and 2005 were genotyped (Table 1).

**Table 1:** Details of the genotyped adult and juveniles Atlantic salmon. The samples include the 876 returning adults caught at the Malbaie River dam from summer 2002 to 2004 and the juveniles caught on the Malbaie R. spawning grounds during spring 2003 to 2005

	Number of individuals perspawning year			
	
	2002	2003	2004	Total
Adults transported above dam	153	324	399	876
Adults used for assignments*	136	287	362	785
Adults with known origin	135	253	348	736
Born in the river (wild origin)	120	167	206	493
Born in the Tadoussac hatchery	15	86	142	243
Stocked at the smolt stage	14	70	51	135
Stocked at the fry stage	1	16	91	108
Returning as SSW	108	204	204	516
Returning as MSW	28	73	154	255
Fry assigned	226	421	494	1141

* Difference between the number of adults used for assignment and the number transported above the dam is due to fish with missing or partial genotypes (see text).

### Molecular analyses

DNA was extracted from fin tissue using the salt extraction method described by Aljanabi and Martinez (1997) and amplified by PCR at eight microsatellite loci: SsaD71, SsaD144, SsaD85, SSaD170 (King et al. 2005), SSa197 (Oreilly et al. 1996), Ssosl417 (Slettan et al. 1995), Sssp2201 and Sssp2213 (Paterson et al. 2004). PCR products were run on an ABI 3100 automated capillary sequencer (Applied Biosystems). Allelic sizes were scored using GENOTYPER™ v.3.7 NT software (Applied Biosystems, Foster City, CA, USA).

### Parental allocation of fry

Parental allocation was conducted using PASOS (Duchesne et al. 2005). PASOS is based on a restricted scoring error tolerance approach that allows optimizing the proportion of correct assignments and estimating the proportion of uncollected parents. The latter was performed using the cumulative allocation option: offspring are allocated sequentially, that is, first using one locus, then two, three, and so forth until a set of allocations is obtained for each subset of *l* = [1… *L*] loci, where *L* is the total number of loci used in the study. This procedure provides a curve of allocation rate as a function of the cumulative number of loci. Allocation rate will decrease as the number of loci increases and will eventually level off to a value corresponding to the proportion of uncollected parents, thus allowing to estimate this parameter from the data (see Duchesne et al. 2005 for details). This was not only useful to interpret the parental allocation rate but also to estimate the contribution of sexually mature resident parr males to reproduction. Indeed, even if we obtained DNA from most anadromous spawning adults in a given year (Table 1), nonmigratory sexually mature parr may contribute to reproduction but will not be sampled at the dam as returning adults (Martinez et al. 2000; Weir et al. 2005; Saura et al. 2008).

We thus conducted parental allocation separately for the 2003, 2004, and 2005 fry samples (produced by spawning adults returning in 2002, 2003, and 2004, respectively). Sexes of individuals were not included in the analysis because of the uncertainly around sex determination using visual criteria for many individuals. To estimate the correct allocation rate, we performed simulations in PASOS using the following settings. These simulations use parental allele frequencies to generate pseudo-offspring that are then re-allocated. For technical and logistic reasons, it was not possible to do twice the procedure of extraction, purification, and amplification on a consequential number of individuals to estimate precisely genotyping error rates. Thus, an error model was chosen, so that we would obtain a conservative estimate of allocation rate. We fixed genotyping errors of one offset (i.e., an error of more or less one tandem repeat) at a 5% rate and errors of two offsets at a 1% rate. These rates were chosen because they are somewhat higher than the upper bound of what is typically observed with microsatellites (Hoffman and Amos 2005; Pompanon et al. 2005). It is important to mention that the error rate entered in PASOS does not affect the allocation of real offspring, but only affects the simulations. Thus, assuming a (too) high error rate is conservative in the sense that it will lead to an underestimation of the expected rate of correct allocation.

Adults with incomplete genotypes were discarded from the analyses because PASOS requires complete genotypes. The number of missing parents for each year (potentially mature parrs and adults with incomplete genotypes) as estimated from cumulative allocation was entered in the simulations. A thousand ‘pseudo-offspring’ were generated from virtual parents created by resampling with replacement of alleles based on empirical frequencies. Ten iterations were performed for each year separately, and the average of correct assignments was used as a point estimate of correctness rate.

### Origin of spawners

To estimate the genetic contribution of stocked salmon to the reproductive output in the Malbaie River, we first classified returning adults from the 2002, 2003, and 2004 spawning runs according to their origin (wild born or hatchery born). Smolt-stocked fish were not adipose fin clipped, but the growth pattern of scales easily allows identifying hatchery-born adults that had been released in the river at the smolt stage (Lund and Hansel 1991; Stokesbury and Lacroix 1997). Identification of fish released at the fry stage was performed by parental allocation with PASOS following the procedure described previously and using the returning adults as offspring to be assigned. For these fish, only the pool of captive breeders from Tadoussac was included as potential parents. We did not estimate the correct allocation rate by simulation as we did for fry because the parents of wild-born returning adults were necessarily unknown. Hence, we could not simulate pseudo-offspring from these unknown parents. Consequently, performing simulations only based on Tadoussac genotypes was not warranted because it would not provide any estimate of correctness for wild-born returning adult neither an estimate of misallocations for hatchery-born spawners. We thus used the following criteria to filter allocations. An adult was considered as stocked from the hatchery at the fry stage if (i) the scale growth pattern did not identify it as released at the smolt stage, (ii) if it was allocated to two Tadoussac (hatchery) parents, and (iii) it had at most one incompatible allele with the identified parents.

The genotypes for the breeders in hatchery were known only for those individuals present at Tadoussac from 2000 onwards. Therefore, adults of hatchery origin returning to spawn in the Malbaie River in 2002 were more likely to remain unallocated than those from 2003 because the former had a higher probability to have been born before 2000, that is, from Tadoussac parents not included in this study. This was also the case for 2003 relative to 2004 spawners. Consequently, it was expected that an increasing proportion of returning adults would be successfully identified as being stocked as fry with increasing year, and this was considered in all subsequent result interpretations.

### Analysis of reproductive success

We used the number of fry caught in the river allocated to a given spawner as a proxy for its reproductive success (RS) in the wild. Although this measure is partial (i.e., not all offspring of a spawner were collected), it allowed estimating the success of hatchery-born fish relative to that of wild-born fish (RRS) by computing the ratio in RS of the former over the latter. Confidence intervals around the RRS of stocked fish for the 3 years pooled were estimated by bootstrapping. As the proportion of repeat spawners is low in Atlantic salmon (e.g., <10%; Fleming and Reynolds 2004) and as there was to our knowledge no study reporting any difference in iteroparity rate between wild-born and hatchery-born fish, the RRS in a given year was considered as proxy for the lifetime RRS.

To further disentangle the factors affecting reproductive success, we used generalized linear models (GLMs) to test for the effect of origin/stocking stage (stockstage, with possible values ‘wild’, ‘fry’, and ‘smolt’), sex, and number of winters spent at sea (seawinter, with possible values ‘ssw’ and ‘msw’ corresponding respectively to only one or several (mainly two) winters at sea). We also controlled for among-year variability in fry sampling effort by including year as a fixed factor. As reproductive success is a count of offspring, the GLMs assumed a Poisson distribution (i.e., we used a log link function). Models were fit using the glm function in R Development Core Team (2012). R: A language and environment for statistical computing. R Foundation for Statistical Computing, Vienna, Austria. ISBN 3-900051-07-0, URL http://www.R-project.org/. We first ran a general model (Model 1) with first-order interactions among seawinter,stockstage and sex. We then fitted two reduced models: a simple additive model (model 2) (i.e., without interactions) including the four variables and a model (model 3) in which we removed sex because its determination by external morphology may not be totally reliable and the sex was in fact uncertain for a number of individuals (Table 1). Models fit to the data were compared by chi-square analysis of deviance.

## Results

### Parental allocation

In 2002, 2003, and 2004, a total of 153, 324, and 399 returning anadromous adults were transported above the dam, respectively. For a majority of them (89, 6%), we obtained a complete genotype that was used for parental allocation (Table 1). Others were adults known to have passed the dam, but for which no sample was available or adults that were discarded because of missing information at one or more locus (PASOS requires complete genotypes for parents). The 1141 fry that were sampled during summer in the Malbaie River between 2003 and 2005 and genotyped were assigned to putative parents that spawned above the dam during the fall between 2002 and 2004. All loci were highly polymorphic, thus all potentially offering high power of parental assignment (Bernatchez and Duchesne 2000). Depending on the locus, observed heterozygosity ranged from 0.86 to 0.95 with a median value of 0.94. The number of allele ranged from 14 to 51 depending on the locus, with a median value of 31. Genotype allocation and simulation with PASOS indicated that about 70% of the breeders of fry caught in 2003 in the Malbaie River were among those from the 2002 spawning run. The expected correct allocation rate was of 93.5%. In the same way, for the 2004 fry cohort, about 75% of parents were sampled in the 2003 run according to the PASOS simulations. However, the correct allocation rate was lower (82%). For the 2005 cohort, about 76% of the parents were sampled in the 2004 run according to simulations, with a correct allocation rate of 84%.

### Assignment power in wild-born versus stocked fish

Before comparing the reproductive success of wild-born vs stocked fish, we needed to ensure that there was no inherent bias favoring one group in the ability to perform parental assignments (Ford and Williamson 2010). For instance, it could be easier to assign offspring born to wild-born parents if the latter exhibited a higher heterozygosity than stocked parents. This was not the case, however, as expected heterozygosity was similar among wild-born and stocked parents, respectively, 0.91 and 0.89. We also conducted simulations in PASOS to assess (i) whether it was easier to assign an offspring born to wild-born parents than an offspring born to stocked parents and (ii) whether the expected assignment error rate was comparable for the two groups (Appendix S1). We found that the difference between the two groups was negligible regarding the first point. Regarding the second, the expected proportion of assignment errors was higher for offspring produced from stocked parents. Similarly, Ford and Williamson (2010) found a lower success of assignment to hatchery-born, relative to wild, Chinook salmon and showed that this lower performance was due to a smaller effective population size in the grandparental generation (i.e., the breeders in hatchery) for the former, as well as to the higher level of relatedness among hatchery-born spawners. In our case, however, the vast majority (89.3%) of errors were predicted to be offspring born to stocked parents misassigned to other stocked parents and thus to have a limited (if any) effect on RRS estimation.

### Contribution of supportive breeding to number of anadromous fish and to reproduction

A total of 173 524 smolt and 221 981 fry were stocked in the Malbaie River from 1998 to 2002, whereas 243 fish caught at the dam originated from the hatchery and 493 were wild-born fish (Table 1). From 2002 to 2004, an increasing proportion of returning adults were assigned to the hatchery pool. This was expected because the absolute contribution of supportive breeding was probably underestimated for the 2002 and 2003 spawning runs relative to 2004 (see Methods). Thus, among returning adults from 2002 to 2004, we identified, respectively, 14, 70, and 51 smolt-stocked and 1, 16, and 91 fry-stocked salmon.

While the proportion of returning adults originating from the hatchery in summer 2002 was 11% (Table 1), their contribution to cohort 2003, calculated as the proportion of assigned offspring–parent dyads involving a hatchery-born parent, was 3.4% (*n* = 298 dyads). These two proportions are significantly different (χ2 = 9.63, *P* = 0.002). Therefore, while the limited contribution of stocked fish largely reflected their small proportion in the river, their relative reproductive success was lower than that of wild-born fish, based on the proportion of spawners of both hatchery and wild origin. The entire contribution of hatchery-born fish was from 14 salmon released as smolt, while only one spawner was released as a fry and did not contribute any offspring, according to PASOS. However, the number of fry-stocked individuals is likely underestimated by our strict assignment criteria (see Methods). Both smolt- and fry-stocked fish returning in 2003 were much more abundant than in 2002, representing 35% of returning adults. However, their relative contribution to reproduction was again significantly lower than expected based on their numbers (19%; χ2 = 24.3, *n* = 568 offspring–parent dyads, *P* < 0.001). The proportion of hatchery-born fish was higher for the parents of the 2005 cohort, yet still significantly below that of wild-born fish (representation = 41%, contribution = 31%; χ2 = 11.2, *n* = 689 offspring–parent dyads, *P* < 0.001). Thus, over the 3 years, the relative reproductive output of the hatchery-born salmon was significantly lower than expected based on their abundance relative to that of wild-born salmon.

### Reproductive success of individual fish

Between 0 and 25 fry were assigned per individual hatchery-reared fish, corresponding to an average reproductive success of 1.28 (Table 2, Figure S1). For wild-born fish, from 0 to 34 fry were assigned per individual, for an average reproductive success of 2.34. Thus, the overall RRS of hatchery-reared fish was 0.55 (CI: 0.35–0.82) when all individuals were pooled. The weighted geometric mean of hatchery-reared fish RRS for the 3 years was 0.53. While the RRS of hatchery-reared fish increased from 2002 to 2004, it remained far below 1 for all years: 0.30 in 2002, 0.45 in 2003, and 0.64 in 2004 (Table 2). Overall, the RRS of fish released as smolt was 0.42 (CI: 0.20–0.74), which was lower than that of fish released as fry, 0.71 (CI: 0.42–1.22) (Table 2). Individual reproductive success of females was about twice higher than that of males for both wild- and hatchery-born fish (Table 2). Also, the reproductive success of SSW individuals was much lower than the RS of MSW individuals, both for wild-born (1.14 vs 4.25, respectively) and hatchery-born fish (0.87 vs 2.77, respectively). The RRS of hatchery-born fish was of 0.76 (CI: 0.48–1.18) for SSW (i.e., when compared with SSW wild-born fish), which was slightly higher than that of MSW fish, namely 0.65 (CI: 0.28–1.19) when compared with MSW wild-born fish. Considering adults returning in 2004, the RRS of hatchery-born fish was on average 0.64, that is, 0.34 for smolt-stocked fish and 0.80 for fry-stocked fish.

**Table 2:** Reproductive success (RS) of Atlantic salmon spawning in the Malbaie River as estimated from parental allocation using PASOS based on microsatellites markers. relative reproductive success (RRS) is the reproductive success of hatchery-reared fish relative to that of wild-born fish belonging to the same category (e.g., SSW, female and fry stocked). A dash indicates that no fish among those sampled entered in a particular category, while ‘n.a.’ means ‘not applicable’. For the RRS of fish for all years pooled, we report the 95% confidence intervals estimated by bootstrapping 10 000 times

Origin	Measur-ement	Year	Time at sea		Sex			Stage stocked		Total
		
			ssw	msw	female	ind	male	fry	smolt
Wild	RS	2002	1.28	5.69	3.82	2.23	1.66	n.a.	n.a.	2.13
		2003	1.12	5.08	4.41	4.48	1.54	n.a.	n.a.	2.58
		2004	1.02	3.41	2.26	2.2	1.42	n.a.	n.a.	2.26
		All years pooled	1.14	4.25	3.49	3	1.53	n.a.	n.a.	2.34
Hatchery	RS	2002	0.71	–	–	0.8	0.67	–	0.67	0.63
		2003	0.81	7.2	4.63	0.81	0.83	1	1.19	1.15
		2004	0.94	2.43	1.3	1.93	1.11	1.81	0.77	1.44
		All years pooled	0.87	2.77	2.1	1.61	0.95	1.66	0.98	1.28
Hatchery	RRS*	2002	0.56	–	–	0.36	0.4	–	0.31	0.3
		2003	0.72	1.42	1.05	0.18	0.54	0.39	0.46	0.45
		2004	0.92	0.71	0.58	0.88	0.78	0.8	0.34	0.64
		All years pooled	0.76 (0.48–1.18)	0.65 (0.28–1.19)	0.6 (0.09–1.51)	0.54 (0.23–1.07)	0.62 (0.36–1.05)	0.71 (0.42–1.22)	0.42 (0.20–0.74)	0.55 (0.35–0.82)

* The RRS of fry- and smolt-stocked fish is relative to all categories of wild-born fish.

**Figure 1:**
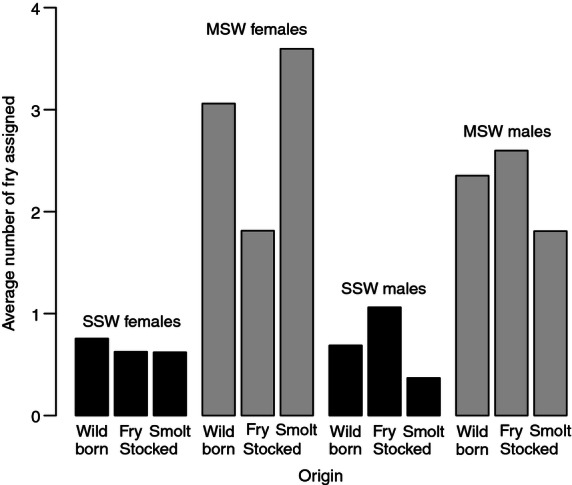
Average number of fry assigned per adult fish as predicted from a generalized linear model (GLM) (our model 1) fitted to salmon data. Predicted values are for the 2004 spawning run, that is, the year when the proportion of adults born in hatchery is best estimated (see text). Values are shown separately for males and females returning after a single year (SSW; black bars) or multiple years (MSW; gray bars) at sea and, within each of these two groups, for wild-born fish, fry-stocked fish, and smolt-stocked fish.

Generalized linear models (GLMs) results are reported in Table 3, and the RS values predicted by the best model for the different groups of fish are shown in Fig. 1. The best model was the general model (1) including the four variables and interactions among seawinter,stockstage, and sex. A purely additive model (2) or the model with sex removed (3) did not fit the data as well (Table 3). In all models, spawning year had a significant effect on reproductive success, reflecting the among-year variation in sampling effort/success. The factor having the most marked effect on reproductive success in all models was seawinter, with SSW salmon performing less than MSW salmon. Importantly, the differences between hatchery-reared and wild-born fish were relatively small within groups defined by sex and seawinter.

**Table 3:** Effects of spawning year, time spent at sea (seawinter), stage at stocking (stockstage), and sex on the reproductive success (RS) of adult Atlantic salmon returning in the Malbaie River to spawn between 2002 and 2004. RS was obtained from parental allocation analysis based on microsatellites and modeled as a Poisson distribution. The middle part shows a model comparison based on a chi-square analysis of deviance. The lower part of the table reports partial regression coefficients (±SE) for each factor tested by fitting generalized linear models (GLMs) to salmon data (*n* = 539 adults). Coefficients were fitted relative to 2002 MSW female fish stocked as fry. A ‘×' denotes a second-order interaction term

	Model 1	Model 2	Model 3
Model description	year + (seawinter + stockstage + sex)^2^ *	year + seawinter + stockstage + sex	year + (seawinter + stockstage)^2^
Deviance	1980.69	2009.9	1995.47
df	527	532	531
*P*	n.a.	<0.001	<0.001
year: 2003	−0.18 (0.09)	−0.13 (0.09)	−0.17 (0.09)
year: 2004	−0.66 (0.10)^***^	−0.65 (0.10)^***^	−0.66 (0.10)^***^
seawinter:ssw	−1.06 (0.32)^***^	−1.24 (0.08)^***^	−0.69 (0.19)^***^
stockstage:smolt	0.69 (0.27)^*^	−0.48 (0.15)^**^	0.34 (0.23)
stockstage:wild	0.52 (0.21)^*^	−0.09 (0.11)	0.26 (0.16)
sex:male	0.36 (0.29)	−0.20 (0.08)^**^	n.a.
ssw × smolt	−0.69 (0.38)	n.a.	−1.33 (0.29)^***^
ssw × wild	−0.34 (0.27)	n.a.	−0.68 (0.21)^**^
ssw × male	0.17 (0.21)	n.a.	n.a.
smolt × male	−1.05 (0.41)^*^	n.a.	n.a.
wild × male	−0.62 (0.30)^*^	n.a.	n.a.

n.a., not applicable.

* 0.05 > *P* > 0.01; ^**^0.01 > *P* > 0.001; ^***^P < 0.001.

† Second-order interactions are included for variables in brackets. For instance, model 1 expands to year + seawinter + stockstage + sex + seawinter × stockstage +seawinter × sex + stockstage × sex.

### Sex ratio and time at sea among wild-born vs hatchery-born fish

**Figure 2:**
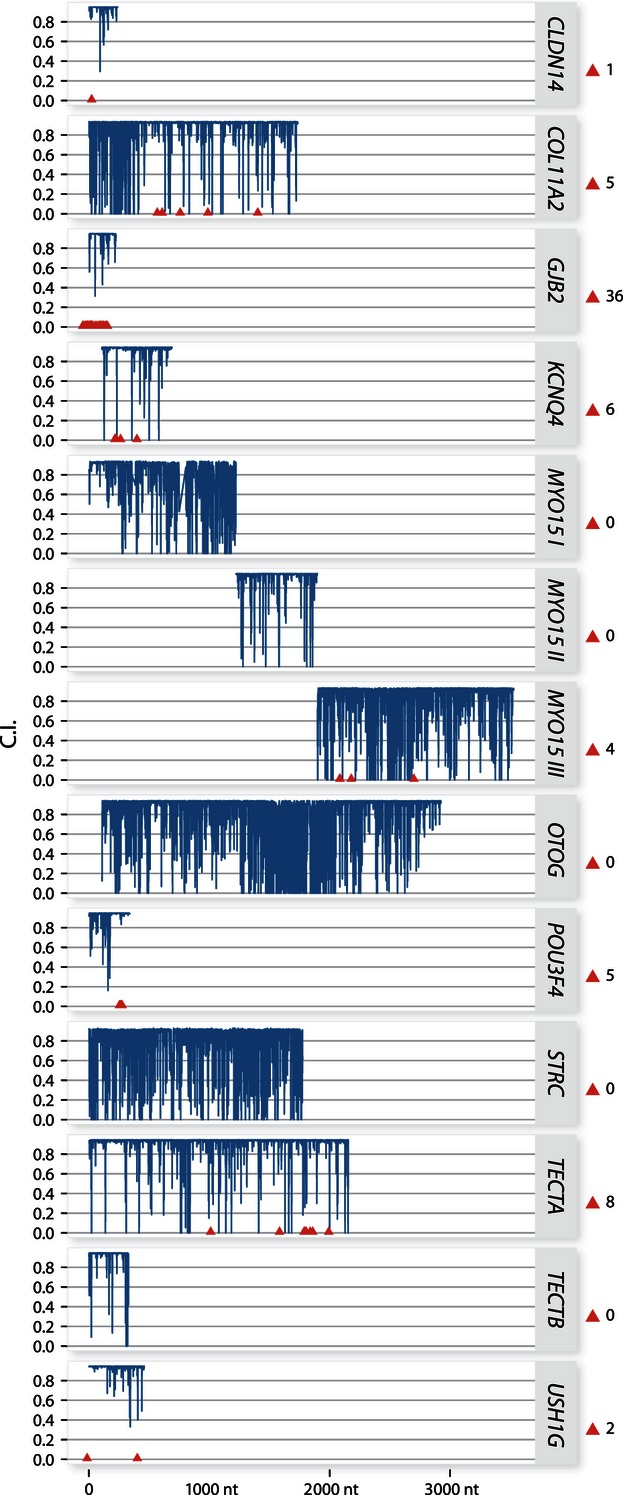
Average sex ratio among adult Atlantic salmon returning to the Malbaie River from 2002 to 2004 after a single winter at sea (SSW), several ones (MSW) or both, and born in hatchery and stocked as fry or smolt or both, or born in the wild. Proportions are given as an average of the 3 years, and error bars indicate standard deviation among years. Values are given for individuals identified as female, male, or uncertain (‘ind’) when there was no clear evidence of the sex of the individual.

Among the wild-born anadromous spawners returning from 2002 to 2004 to the Malbaie River, and for which the sex was confidently estimated, 25% were identified as females and 75% as males (Fig. 2). These percentages differ according to time spent at sea, with SSW and MSW wild-born fish including 91% and 48% of males, respectively (χ2 = 154.05, *P* < 0.0001). No significant difference of sex ratio was found among wild-born, and smolt- and fry-stocked fish, either for SSW or for MSW fish.

**Figure 3:**
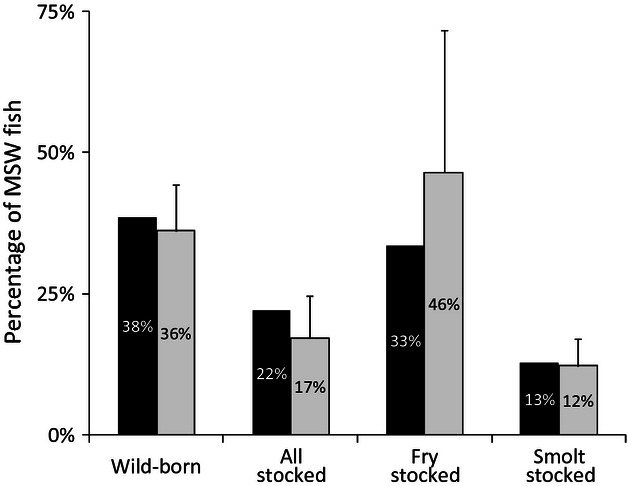
Percentage of multi-sea-winter individuals among adult Atlantic salmon born in the wild or in hatchery and that returned to spawn in the river Malbaie between 2002 and 2004. For captive-bred fish, values are also reported separately for those stocked as fry (Fry) and as smolt (Smolt). Percentages are for all years pooled (dark bars) or as averages of the 3 years (gray bars, ± SD).

The percentage of MSW fish was significantly lower among hatchery-reared individuals than among wild-born ones (38.5% of wild-born, 21.8% of hatchery-born, χ^2^ = 21.4, *P* < 0.001; Fig. 3). Especially, the percentage of MSW fish was lower among smolt-stocked fish (12.7%) than among fry-stocked (33.3%) and wild-born fish (38.5%). When considering spawning years separately, the difference was significant for 2003 (37.1% among wild-born fish, 5.3% among hatchery-reared ones, χ^2^ = 32.2, *P* < 0.001) and 2004 (51.8% among wild-born fish, 33.6% among hatchery-reared ones, χ^2^ = 11.8, *P* < 0.001). The percentage of MSW was similar for both groups in 2002 (19% of wild born, 13% of hatchery, χ^2^ = 0.4, *P* = 0.51), that is the year when the representation of hatchery fish was likely the most underestimated. Among SSW fish, those stocked as fry had a higher RRS than fish stocked as smolt (respectively, 1.11 and 0.54), while we observed the inverse pattern for MSW fish (respectively, 0.58 and 0.81).

## Discussion

In this study, we used molecular parental assignment to infer the reproductive success of hatchery-born anadromous Atlantic salmon released in the Malbaie River in Québec after a single generation in captivity and returning to spawn after their journey at sea. Over three consecutive spawning years, these fish had a significantly lower RS than wild-born salmon, a reduction in a large part attributable to the modification of their life history. Specifically captive-bred salmon returned in a greater proportion than wild-born salmon after a single winter at sea and single-sea-winter had a lower RS than multi-sea-winter fish. However, the drop in RS was lower for fry-stocked than for smolt-stocked fish, suggesting a greater negative impact of the early life in hatchery on the later.

## Reduced reproductive success of hatchery-reared Atlantic salmon in the wild

The lower reproductive success of captive-bred Atlantic salmon *in natura* relative to that of their wild-born conspecifics corroborates the results obtained for steelhead (*Oncorhynchus mykiss*) (Araki et al. 2007b), chinook salmon (Williamson et al. 2010), and coho salmon (*O. kisutch*) (Thériault et al. 2011). Therefore, for these four salmonids species, a single generation of hatchery rearing of local fish was sufficient to decrease the RS of individuals released in the wild. Other studies also showed that domestic Atlantic salmon escaped from commercial farms had a reduced RS in the wild (Fleming et al. 2000; McGinnity et al. 2003). Here, we uncovered RRS for stocked fish (0.55) similar to values reported by Araki et al. (2007b) for steelhead (0.55) and Thériault et al. (2011) for coho salmon (RRS ranging from 0.56 to 1.11). This level of RRS is, however, higher than that for escaped domestic Atlantic salmon (0.24 for females and 0.32 for males; Fleming et al. 2000), which are likely more exposed to selection and environmental carryover effects of captive life than stocks from local genetic origin used in supportive breeding programs, especially when the latter involve a single generation in captivity (Araki et al. 2008).

## Sex ratio and reproductive success

Our results showed a higher RS for females than for males, but a similar decrease in RRS for hatchery-reared males and females. It is consistent with the fact that the sex ratio of spawning Atlantic salmon is usually male biased, with several males competing for the fertilization of the eggs from each female (Fleming 1996, 1998; Fleming and Reynolds 2004). Accordingly, we found a male-biased sex ratio among anadromous returning adults, with males representing on average from 55% to 86% of the individuals (the uncertainty originating from the fact that the sex of 31% of fish was not confidently determined). Moreover, several studies reported that variable proportions of eggs could be fertilized by early maturing freshwater male parr (Bohlin et al. 1986; Martinez et al. 2000; Garant et al. 2003; Weir et al. 2005; Saura et al. 2008), which could increase the bias in sex ratio among breeders and the male–male competition and further decrease the reproductive success of anadromous males. The fact that we did not find a noticeable difference between the RRS of hatchery-reared males and females suggests that captive breeding may affect males and females to a similar degree, as found by Araki et al. (2007b) but in contrast with Fleming et al. (2000), Williamson et al. (2010), and Thériault et al. (2011).

## Proportion of single- and multi-sea-winter fish and reproductive success in smolt-stocked fish

Our study underscores that the differences in reproductive success between wild-born and captive-bred salmon were mostly driven by the larger proportion of smolt-stocked returning as SSW and, to a lesser extent, by the poorer performance of captive-bred fish within each migratory class. Given that MSW are typically larger than SSW, this is in line with studies showing that salmon breeding success is positively correlated with body size and thus the number of winters at sea (Fleming 1996). The higher proportion of SSW among smolt-stocked fish than among fry-stocked or wild-born fish may originate from a higher growth rate of juveniles in hatchery than in the wild and be responsible for plastic modification of migration tactics (Naevdal et al. 1978; Kallio-Nyberg and Koljonen 1997; Jonsson and Jonsson 2006; Ford et al. 2012). On the other hand, an increasing number of studies have reported a reduced survival of stocked fish (Ford and Myers 2008 and Araki and Schmid 2010) that could result in a decrease in the proportion of MSW fish. In the Malbaie River, estimates of the abundance and origin of smolt migrating to sea in 2002 and the abundance and origin of adult salmon entering the River 1 year later revealed that the return rate of salmon stocked as smolt (0.36%) was 6–7 times lower than that of naturally produced smolt (2.4%) (Auclair et al., unpublished data). Furthermore, the marine growth of SSW salmon derived from stocked smolt was significantly less than that of SSW salmon derived from naturally produced smolt (Auclair et al., unpublished data). Other studies have shown that stocked salmon tend to spend fewer winters at sea than wild-born salmon as a potential result of either carryover effects of the hatchery environment or selection (Kostow 2004; Jonsson and Jonsson 2006; Kallio-Nyberg et al. 2011; but see McGinnity et al. 2003). As time spent at sea correlates with body size, which in turn correlates with reproductive success, the reduction in time at sea appears to play an important role in reducing the fitness of the fish stocked in the Malbaie River.

## Life-history trade-offs and relative reproductive success (RRS) in hatchery-born fish

Trade-offs between the time spent growing at sea, survival, and fecundity could be involved in the RRS pattern observed in particular for smolt-stocked fish. Growth rate and body size are heritable traits in salmon (e.g., Riddell et al. 1981), and a genetic correlation was uncovered between sexual maturation and anadromous migration in Atlantic salmon (Paez et al. 2011). Consequently, a lower survival of smolt-stocked fish may favor a SSW strategy at the cost of a lower fecundity.

Alternatively, a greater growth rate of juveniles in hatchery than in the wild could result in a plastic response in migration tactics (Naevdal et al. 1978; Kallio-Nyberg and Koljonen 1997; Jonsson and Jonsson 2006). This also agrees with life-history theory predicting a negative correlation between growth rate and age at maturity (Stearns and Koella 1986). A study by Jokikokko et al. (2006) suggest a reduced survival of smolt-stocked Atlantic salmon, which likely affects the optimal age of maturation and migration in view of life-history trade-offs discussed previously. The survival of smolt could be reduced by a poor timing of release (Chittenden et al. 2010). Carryover effects of an environmentally impoverished hatchery environment may modify phenotypes and consequently the fitness of wild populations by affecting risk-taking behavior (Roberts et al. 2011), feeding capability (Rodewald et al. 2011), or through negative interactions among wild-born and stocked fish (e.g., competitive or aggressive behavior) (Fleming et al. 2000; Jonsson and Jonsson 2006, 2009). Importantly, Blanchet et al. (2008) found that juvenile born in hatchery and stocked in the Malbaie River were more aggressive and responded differently from their wild counterparts to competition.

## Reproductive success in fry-stocked salmon

The lower reproductive success of fry-stocked fish in spite of proportions of SSW/MSW similar to those in wild-born fish could originate from a modified selective regime in captivity. Viability selection can occur during early life feeding (Elliott 1989). If early mortality is lower in the hatchery, then this might also relax selection on genes involved in fecundity. Moreover, limited sexual selection in hatchery may have a similar effect on fry-stocked fish (Wedekind 2002; Neff et al. 2008).

## Limitations of the study

While only 10.4% of the parental anadromous genotypes were incomplete and thus not used in PASOS parental assignments, between 25% and 30% of parent-fry dyads involved uncollected parents, in other words offspring assigned either to one collected + one uncollected parent (37–42% depending of the year) or to two uncollected parents (3–11%). This result may be consistent with a non-negligible participation of precocious parr to reproduction. Indeed, these males can represent a large proportion (up to 60%) of the male breeders in Atlantic salmon populations (Hutchings and Jones 1998). Here, the number of missing parents might have been slightly over-estimated in our simulations. In PASOS cumulative allocation procedure, the estimation of the number of missing parents decreases as loci are added to the analysis, until it levels off to a lower bound that should be the correct estimation. Thus, we cannot exclude that adding one or two loci would have lead to slightly lower estimations of missing parents. In addition, PASOS does not allow genotyping error models with more than two offsets. Such errors (e.g., larger allele dropout) will typically cause allocations to an uncollected parent, and there is no reason why they should be more frequent in one group of spawners (wild or stocked) than the other. Consequently, these errors will mostly result in a loss of power (increased allocations to uncollected parents).

A source of uncertainty that could affect our conclusions is the proportion of fry-stocked spawners estimated for each year. As explained previously, this proportion is likely less underestimated from 2002 to 2004. Nevertheless, the performance of the captive-bred fish is lower than that of wild-born parents for the 3 years. Another source of uncertainty is around the assumption that wild-born spawners had themselves wild parents. Actually, some of their parents (hence grandparents of the fry that we sampled) could had been stocked in the Malbaie River in the 1990s. Consequently, the baseline comparison may not be purely with wild fish, thus leading to an overestimation of the RRS of stocked fish. However, we do not consider this as a major issue in the current context because our main objective was to specifically focus on the effect of captive breeding over one generation and because any additional effect owing to the captivity of some ancestors of wild-born spawners will likely introduce noise and thus reducing the apparent difference between wild and captive-bred fish.

## Evolutionary and practical consequences of supporting breeding on wild populations

The use of supportive breeding programs is widespread but nonetheless controversial (Aprahamian et al. 2003; Araki and Schmid 2010). For instance, a recent study on steelhead demonstrates that only one generation in captivity can result in a substantial response to selection on traits that are beneficial in captivity but maladaptive in the wild (Christie et al. 2012). Our results raise several issues regarding the conservation of wild salmonid populations.

First, as a large part of the performance reduction in stocked salmon appears to originate either from carryover effects or from selection during hatchery life on smolt-stocked fish, this study supports previous recommendations to limit the time spent by individuals in captivity (Frankham 2008; Fraser 2008; Williams and Hoffman 2009; Araki and Schmid 2010). To this end, it is noteworthy that the provincial authority in charge of managing Atlantic salmon in Québec (Ministère des Ressources Naturelles et de la Faune) has recently abandoned stocking salmon at the smolt stage and continue to release salmon at fry stage. However, selection and carryover effects could still occur at younger life stage, affecting fry-stocked fish performances in the wild. In that matter, our study also supports propositions in favor of more natural rearing conditions for captive juveniles (Blanchet et al. 2008; Kallio-Nyberg et al. 2011; Rodewald et al. 2011) or for their release at a younger age, such as unfed fry (Metcalfe et al. 2003; Thériault et al. 2011). Shortening the life in hatchery may furthermore increase the survival of stocked fish (Rideout and Stolte 1988; but see Thériault et al. 2010) but may also limit the cost of such operations.

Second, it is recognized that maintaining a relatively small number of native captive breeders may on the long term result in a decrease in the effective population size and genetic diversity of the stocked population (Wang and Ryman 2001; Hansen et al. 2000; Blanchet et al. 2008; but see Gow et al. 2011). Although heterozygosity in hatchery-born spawners was not lower than that in wild-born Atlantic salmon population from the Malbaie River, without examining older samples (prior to 1990), we cannot totally exclude that long-term stocking has reduced the genetic diversity in the river, as shown in coho salmon Eldridge et al. (2009). Besides, the relatively low returns of smolt-stocked fish in the Malbaie River may not only reflect a high mortality but also dispersal in other wild populations (Jonsson et al. 2003), which would modify the local genetic makeup elsewhere (Perrier et al. 2011).

Third, supportive breeding can have a cumulative negative effect on the reproductive success of spawners via introgressive hybridization of hatchery and wild stocks (Fleming et al. 2000; McGinnity et al. 2003; Araki et al. 2007a, 2009; Roberge et al. 2008). As already mentioned, this may have led to an overestimation of the RRS of stocked fish. Such introgressive hybridization could moreover explain why the population has still not recovered to its suitable conservation size (estimated to approximately 1400 adult spawners). Since the beginning of supportive breeding program in the Malbaie River, the increase in the total number of breeders has been modest in previous years (on average 279 spawning adults between 1997 and 2007; data available at http://www.mrn.gouv.qc.ca/publications/faune/bilan-saumon-2011.pdf), although it has increased in more recent years (on average 818 individuals between 2008 and 2011). Overall, this study suggests that the potential negative evolutionary consequences of repetitive supportive breeding on the managed population should be carefully considered before applying this management strategy as a long-term viable option.

## Data archiving statement

Raw data for this study are available in Dryad: doi:10.5061/dryad.4k739.

## Supporting Information

**Figure S1**. Histogram of the number of wild-born progeny assigned per adult individual caught at the dam. **Table S1**. Number of fish annually stocked within the Malbaie R. (at the egg, fry and smolt stages); spawning run and conservation threshold reached (estimated by the Minister of Natural Ressources and Wildlife, Québec) ? = non available. **Appendix S1**. We conducted additional simulations in PASOS to assess whether i) it is easier to assign an offspring born to wild-born parents than an offspring born to stocked parents; ii) different patterns of assignment errors for the two groups could bias our conclusions about the RRS of captive-bred adults returning to the Malbaie River.
