# Supplementary material for: Reduced fitness of Atlantic salmon released in the wild after one generation of captive breeding
Source: Evol Appl. 2012 Nov 22;6(3):472–85. doi: 10.1111/eva.12028 (PMC3673475; doi:10.1111/eva.12028)
Supplement: Supplementary file 1 — Figure S1. Histogram of the number of wild-born progeny assigned per adult individual caught at the dam. Table S1. Number of fish annually stocked within the Malbaie R. (at the egg, fry and smolt stages); spawning run and conservation threshold reached (estimated by the Minister of Natural Ressources and Wildlife, Québec) ? = non available. Appendix S1. We conducted additional simulations in PASOS to assess whether i) it is easier to assign an offspring born to wild-born parents than an offspring born to stocked parents; ii) different patterns of assignment errors for the two groups could bias our conclusions about the RRS of captive-bred adults returning to the Malbaie River. [file eva0006-0472-sd1.docx]

***Supporting information***

**Table S1** Number of fish annually stocked within the Malbaie R. (at the egg, fry and smolt stages); spawning run and conservation threshold reached (estimated by the Minister of Natural Ressources and Wildlife, Québec). ? = non available.

|  |  | 1992 | 1993 | 1994 | 1995 | 1996 | 1997 | 1998 | 1999 | 2000 | 2001 | 2002 | 2003 | 2004 | 2005 | 2006 | 2007 | 2008 | 2009 | 2010 | 2011 |
| --- | --- | --- | --- | --- | --- | --- | --- | --- | --- | --- | --- | --- | --- | --- | --- | --- | --- | --- | --- | --- | --- |
| Stocking | Egg | 118,249 | 15,330 |  |  |  |  |  |  |  |  |  |  |  |  |  |  |  |  |  |  |
|  | Fry |  | 200,000 | 195,000 | 95,013 | 113,260 | 65,235 |  | 49,500 | 80,000 | 27,330 | 65,151 | ? | 63,633 | 87,243 | 51,716 | 107,558 | 88,133 | 62,477 | 95,575 | 137,473 |
|  | Smolt |  |  |  | 50,276 | 51,722 | 48,401 | 47,434 | 35,781 | 24,105 | 33,693 | 32,511 | ? | 55,729 |  |  |  |  |  |  |  |
| spawning run  (before fishing) | | ? | ? | ? | ? | ? | 392 | 390 | 289 | 120 | 117 | 169 | 365 | 420 | 176 | 289 | 337 | 1,032 | 701 | 684 | 854 |
| Conservation  treshold | | ? | ? | ? | ? | ? | 29% | 30% | 17% | 7% | 7% | 10% | 26% | 36% | 15% | 18% | 18% | 67% | 64% | 49% | 55% |

**Text S1 Assignment performance in wild-born vs. stocked fish**

We conducted additional simulations in PASOS to assess whether i) it is easier to assign an offspring born to wild-born parents than an offspring born to stocked parents; ii) different patterns of assignment errors for the two groups could bias our conclusions about the RRS of captive-bred adults returning to the Malbaie River. We used the 2004 spawning run (2005 offspring cohort), i.e. the year for which the proportion of wild-born vs. stocked breeders is the best estimated because fry-stocked spawners were better detected (see main text). Among adults returning that year, 206 were born in the wild and 142 in hatchery (Table 1).

To address the question (i), we conducted independent simulations for the two groups of adults. Thus we used all the 142 captive-bred salmon on the one hand. On the other hand we resampled the 206 wild-born adults to create ten subsets of 142 fish each to level off sample size differences between wild- and hatchery- born fish. We then ran PASOS simulations under the same settings as described in the main text, except that the number of uncollected parents was set to zero, and compared the results for wild-born and captive-bred fish.

To address the second question, we generated 10,000 virtual offspring from wild-born parents (i.e. using only those parents in the analysis) and 10,000 other offspring from captive-bred parents and reassign them to the total pool of parents in our empirical data (i.e. wild-born + hatchery + uncertain origin). We then compiled how many offspring of each type (i.e. either born to wild-born or captive-bred parents) were assigned to each type of parent.

Results for the first set of simulations show that the proportion of assignments to a collected (known) parent is very similar for wild-born (average = 99.54% ± 0.04% SD for the 10 simulations) and hatchery-born adults (98.16%). Therefore, a given offspring is not more likely to remain unassigned (or, equivalently, assigned to uncollected parents) if it was born to captive-bred parents. However, in spite of these similar proportions of assignments, more errors are expected for offspring born to captive-bred parents (15.9% vs. 1.1% for wild-born parents). However, as we show next this difference is unlikely to bias the estimation of RRS for that group.

Results for the second set of simulations indicate that 98.7% of assignments (i.e. of 20,000 parent-offspring dyads) involve a wild-born parent when the virtual offspring was simulated from the pool of wild-born parents and only 0.4% involve a captive-bred parent. Comparatively, 95.3% of assignments involve a captive-bred parent when the offspring was simulated from the pool of captive-bred parents while 1.7% involve a wild-born parent. Remaining cases involve either uncollected parents or of uncertain origin.

Overall, these results show that offspring born to captive-bred parents are not more likely to be unassigned than those from wild-born parents. However, they are more likely to be assigned to a wrong collected parent. Nevertheless, except for a small minority, the wrong assignments will be to other captive-bred parents. One explanation is that stocked salmon being produced from a limited number of breeders in the Tadoussac hatchery, perhaps a higher proportion of them exhibit close relationships (e.g. sibs, cousins, etc) making the assignment more difficult. Anyhow, the difference in misassignments to the wrong group between wild-born and stocked fish (0.4 vs. 1.7%) is clearly too small to create a bias that would explain an overall RRS as low as 0.55 for stocked parents.

**Figure S1 Histogram of the number of wild-born progeny assigned per adult individual caught at the dam.**
